# Sequential Amiodarone‐Induced Thyroid Dysfunction: From Myxedema Coma to Late‐Onset Type 1 Thyrotoxicosis After Drug Withdrawal

**DOI:** 10.1002/ccr3.72158

**Published:** 2026-02-23

**Authors:** Daniela M. Soares, Lia Ferreira

**Affiliations:** ^1^ Endocrinology Department – Unidade Local de Saúde de Santo António Porto Portugal

**Keywords:** amiodarone‐induced hypothyroidism, amiodarone‐induced thyroid dysfunction, amiodarone‐induced thyrotoxicosis, myxedema coma

## Abstract

Our report underscores three key points: (1) the prolonged metabolic effects of iodine load and tissue toxicity even after amiodarone cessation, (2) the possibility of reversible amiodarone‐induced hypothyroidism preceding subsequent thyrotoxicosis, and (3) the need for long‐term thyroid function monitoring, even after amiodarone withdrawal.

## Abstract (pt)

Amiodarone therapy may induce thyroid dysfunction in approximately 20% of patients, either amiodarone‐induced hypothyroidism (AIH) or thyrotoxicosis (AIT). Both situations may occur at any time during treatment or even months to years after drug withdrawal due to tissue storage with slow systemic release. The sequential occurrence of severe AIH followed by late‐onset AIT, particularly type 1, is exceptionally uncommon. We report the case of a 76‐year‐old woman, with long‐standing amiodarone therapy for atrial fibrillation who presented in the emergency department with lethargy, hypothermia, bradycardia, respiratory and renal failure, and severe biochemical hypothyroidism. A Popoveniuc score of 90 points supported the diagnosis of myxedema coma, and intravenous levothyroxine plus hydrocortisone therapy was initiated. She presented favorable evolution with all dysfunctions' resolution and was discharged under 150 μg levothyroxine; amiodarone was discontinued after Cardiology's assessment. Thyroid function normalized under gradually reduced levothyroxine dosing, which was ultimately suspended fourteen months later. Thirty‐two months after amiodarone suspension, the patient developed persistent biochemical hyperthyroidism with negative thyroid autoimmunity. ^99m^Tc‐pertechnetate scintigraphy showed diffuse uptake, compatible with type 1 AIT. Methimazole therapy was initiated, and the patient presented a gradual recovery of thyroid function. This case illustrates the full spectrum of amiodarone‐induced thyroid dysfunction, from life‐threatening AIH to late‐onset type 1 AIT long after drug withdrawal. It underscores the prolonged metabolic consequences of amiodarone exposure, the possibility of sequential opposing thyroid dysfunction states, and the necessity for extended surveillance even years after cessation.

## 1 Introduction

Amiodarone is a class III antiarrhythmic agent, widely used in the management of arrhythmias. It contains a high iodine content—approximately one‐third of its molecular weight—and each 100 mg dose releases about 3 mg of free iodine into circulation, far exceeding normal daily requirements [1, 2]. Owing to its marked lipophilicity, amiodarone accumulates extensively in adipose tissue, skeletal muscle, and the thyroid gland, and has a prolonged elimination half‐life of roughly 100 days. As a result, its toxic effects may emerge or persist long after the drug has been discontinued [2, 3].

Although most patients remain euthyroid due to the Wolff–Chaikoff effect of inhibition of thyroid hormone synthesis, those with underlying thyroid disease may display impaired iodine autoregulation, leading to either iodine‐induced hypothyroidism or hyperthyroidism [4, 5, 6]. Amiodarone also exerts direct toxicity in thyroid follicular cells, which may trigger destructive thyroiditis with the release of pre‐synthesized hormones, even in healthy thyroid glands [7].

Due to these mechanisms, amiodarone‐induced thyroid dysfunction (AITD) occurs in 15%–20% of amiodarone‐treated patients and comprises a continuum including both amiodarone‐induced hypothyroidism (AIH) and amiodarone‐induced thyrotoxicosis (AIT) [8]. Two major forms of AIT are described: type 1 AIT, an iodine‐induced hyperthyroidism typically arising in abnormal thyroid glands, and type 2 AIT, a destructive thyroiditis usually occurring in previously normal glands. Both hypothyroidism and thyrotoxicosis may occur at any time during therapy and even after drug withdrawal, due to tissue storage with slow systemic release [9]. Nevertheless, the occurrence of AIH and AIT in the same patient or myxedema coma development in this context is rare. While a few cases of sequential thyroid dysfunction have been described [9, 10, 11], the specific progression from severe AIH to late‐onset type 1 AIT long after drug withdrawal has not, to our knowledge, been previously reported in the literature.

We report a rare case of sequential AITD beginning with severe AIH complicated by myxedema coma, followed by late‐onset type 1 AIT more than two years after amiodarone withdrawal.

## 2 Clinical Case Presentation

A 76 year‐old woman was admitted to the emergency department with a one‐week history of lethargy, dyspnea, orthopnea, and lower‐limb edema. Her medical background included type 2 diabetes; hypertension; dyslipidemia; valvular heart disease; and persistent atrial fibrillation treated for nine years with warfarin and amiodarone (200 mg twice daily). She denied any personal or family history of thyroid disease, neck radiation, recent exposure to iodinated contrast agents, or corticosteroid use. Physical examination revealed hypothermia (34°C), hypotension (84–94/58–69 mmHg), and bradycardia (45 bpm), as well as decreased breath sounds and peripheral pitting edema bilaterally. Further work‐up reported atrial fibrillation with slow ventricular response, respiratory failure [pCO2 57.1 mmHg (35–45); pO2 68.0 mmHg (80–100)], acute kidney injury [urea 175 mg/dL (10–50) (SI 29.1 mmol/L); creatinine 3.2 mg/dL (0.5–0.9) (SI 282.9 μmol/L)] and severe hypothyroidism with markedly elevated thyroid‐stimulating hormone (TSH) and suppressed free thyroxine (fT4) and free triiodothyronine (fT3). Thyroid autoantibodies were negative (Table 1). A Popoveniuc score of 90 points supported the diagnosis of myxedema coma (Table 2) [12]. The patient was started on intravenous levothyroxine and hydrocortisone therapy, then treated with non‐invasive ventilation and diuretics. Her clinical course improved steadily; hydrocortisone was discontinued after adrenal insufficiency was excluded, and she was discharged on 150 μg levothyroxine. After Cardiology assessment, amiodarone therapy was replaced with bisoprolol.

**TABLE 1:** Laboratory findings since emergency room admission.

	Reference range	T0	T14	T20	T32	T38	T43	T50
Cortisol	6.2–19.4 μg/dL	19.3	—	—	—	—	—	—
TSH	0.30–3.94 mUI/L	**71**	**0.05**	0.55	**0.29**	**0.07**	**0.30**	3.60
Free T4	0.95–1.57 ng/dL	**0.42**	**1.89**	1.52	**1.62**	**1.75**	**1.58**	1.32
Free T3	2.42–4.36 pg/mL	**1.10**	3.88	—	—	2.54	—	—
Anti‐TPO antibody	< 34 UI/mL	< 9	< 9	11.9	—	—	—	12.5
Anti‐Tg antibody	< 115 UI/mL	22.6	13.1	14.3	—	—	—	22.4
TRAb	< 1.22 U/L	—	—	—	—	1.12	—	0.90
TSI antibody	< 0.10 IU/L	—	—	—	—	< 0.10	—	< 0.10
Creatinine	0.5–0.9 mg/dL	**3.17**	**1.23**	**1.13**	**1.37**	**1.47**	—	—
Sodium	135–145 mmol/L	**127**	144	143	145	144	—	—
C‐reactive protein	< 5.0 mg/L	**23.88**	—	—	—	—	—	—

*Note:* SI units converter: cortisol (nmol/L) = (μg/dL)*27.60; creatinine (μmol/L) = (mg/dL)*88.4; free T4 (pmol/L) = (ng/dL)*12.87; free T3 (pmol/L) = (pg/mL)*1.54. Bold values highlight altered laboratory findings regarding reference ranges.

Abbreviations: T: time (in months) since emergency room admission (T0); Tg: thyroglobulin; TPO: thyroid peroxidase; TRAb: thyrotropin receptor antibody; TSI: thyroid stimulating immunoglobulin; TSH: thyroid‐stimulating hormone.

**TABLE 2:** Diagnostic scoring system of Popoveniuc [12].

		Points
**Thermoregulatory dysfunction (°C)**	**34**	**10**
**Central nervous system effects**	**Lethargic**	**10**
**Gastrointestinal dysfunction**	Absent	0
**Precipitating event**	Absent	0
**Cardiovascular dysfunction**		
Bradycardia (bpm)	**45**	**20**
Atrial fibrillation	**Yes**	**10**
Hypotension	**Yes**	**20**
**Metabolic disturbances**		
Hyponatremia (mmol/L)	**127**	**10**
Hypercarbia (mmHg)	**57.1**	**10**
**Total**		90

*Note:* Bold values highlight altered parameters (above 0) regarding the diagnostic scoring system of Popoveniuc.

## 3 Differential Diagnosis, Investigations, and Treatment

**FIGURE 1:**
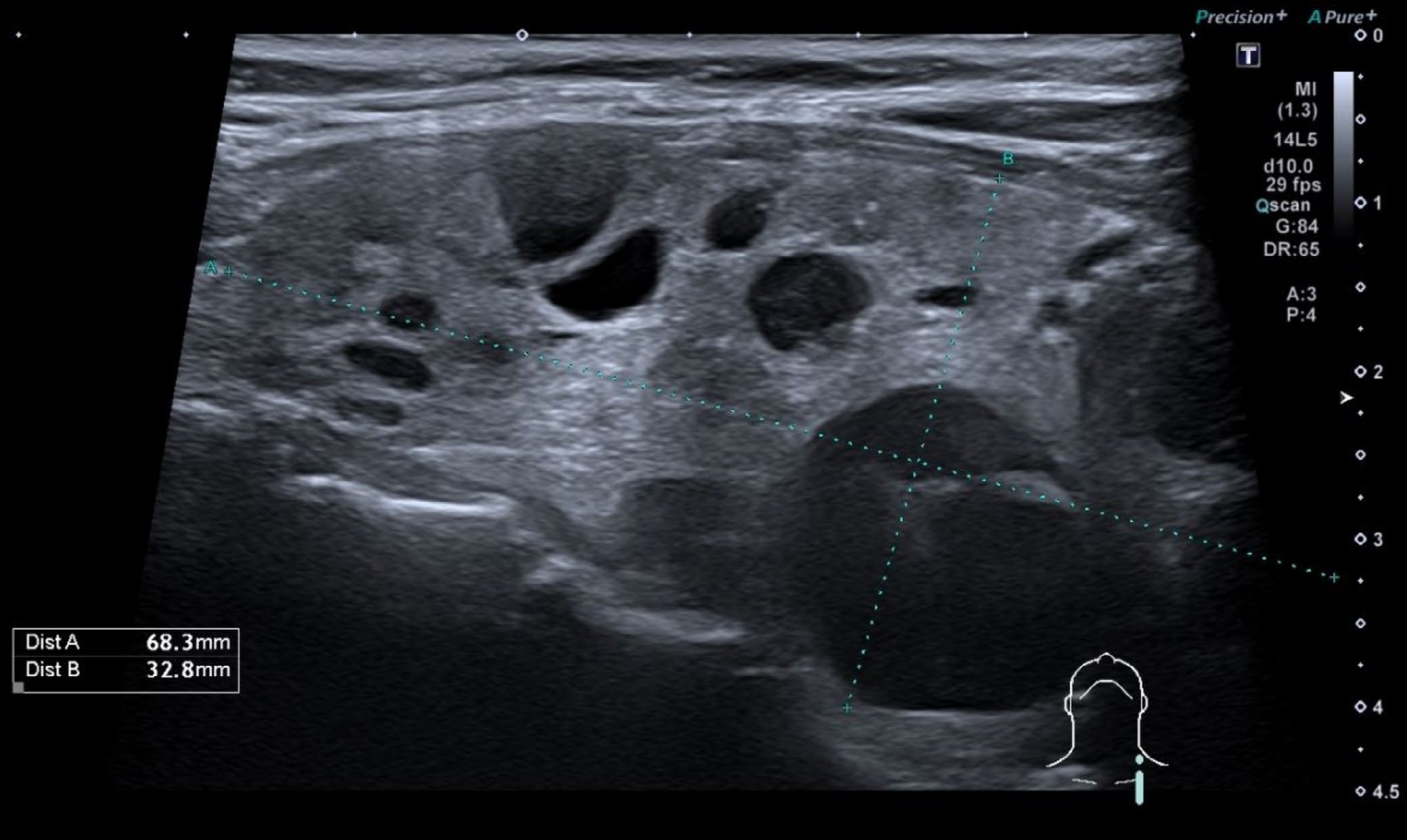
Thyroid ultrasound (longitudinal view); enlarged thyroid gland due to multiple cysts (largest of 23 mm).

**FIGURE 2:**
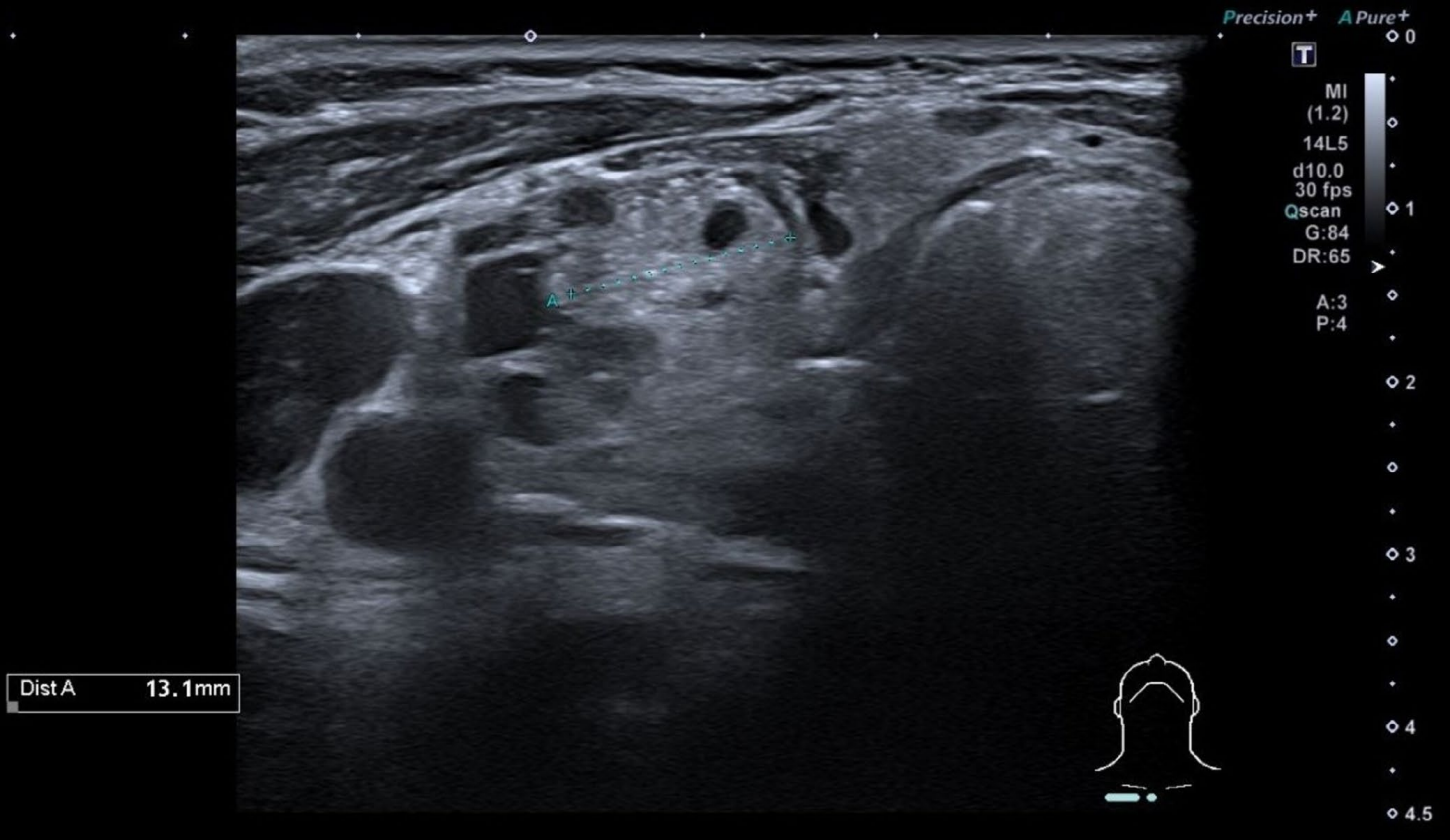
Thyroid ultrasound (transverse view); an isoechogenic solid nodule of 13 mm (EU‐TIRADS 3).

Thyroid ultrasound revealed multiple cysts and a 13 mm isoechogenic solid nodule (EU‐TIRADS 3), and AIH was assumed (Figures 1 and 2). The patient became gradually euthyroid, with progressively lower levothyroxine needs. Fourteen months after the initial presentation, levothyroxine was suspended (Table 1).

**FIGURE 3:**
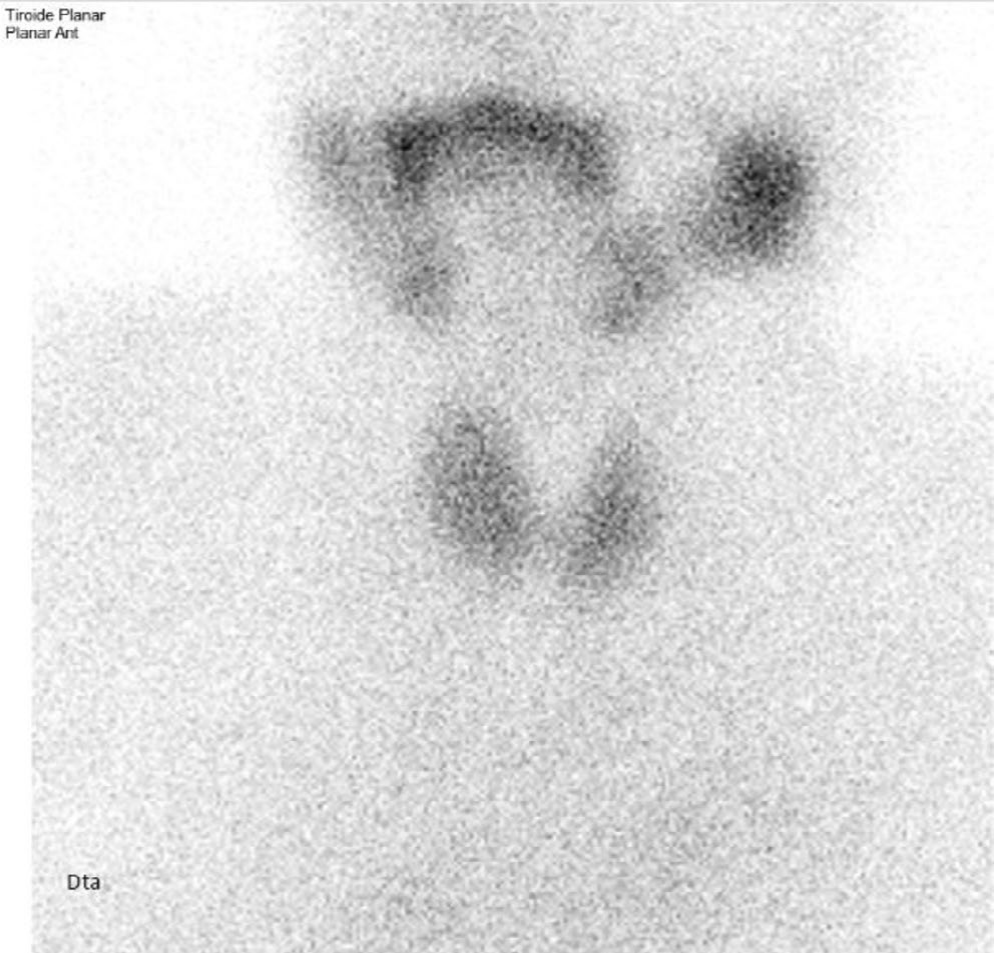
^99m^Tc‐pertechnetate thyroid scintigraphy (coronal view); globally preserved uptake with no hyperactivity areas suggestive of focal hyperfunction (report highlights possible interaction of amiodarone in uptake pattern up to 2 years after withdrawal).

Eighteen months after levothyroxine suspension (thirty‐two months after amiodarone suspension), persistent biochemical hyperthyroidism was identified despite the absence of symptoms and negative thyroid autoimmunity (Table 1). The patient denied exposure to iodinated supplements/contrasts, corticosteroids, or reintroduction of amiodarone therapy. ^99m^Tc‐pertechnetate‐scintigraphy revealed globally preserved uptake without hyperactivity areas suggestive of focal hyperfunction; the nuclear medicine report highlighted the possible interaction of amiodarone in the uptake pattern up to 2 years after withdrawal (Figure 3)—TSH 0.29 mUI/L (0.30–3.94) and fT4 1.62 ng/dL (0.95–1.57) (SI 22.5 pmol/L) at the time of the scan.

**FIGURE 4:**
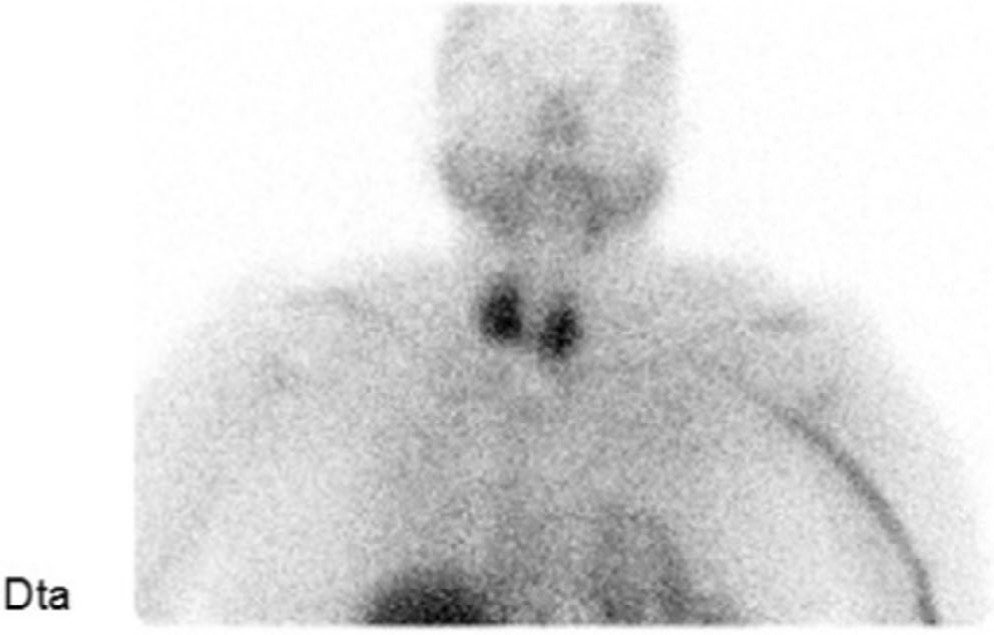
Repetition of ^99m^Tc‐pertechnetate thyroid scintigraphy (coronal view); diffuse increased uptake, compatible with type 1 AIT.

Note: all thyroid function tests and autoantibodies were measured by automated chemiluminescence immunoassays (Siemens Healthineers) using *Atellica IM* assay kits.

She remained clinically euthyroid, but persistent biochemical hyperthyroidism [TSH 0.07 mUI/L; fT4 1.75 ng/dL; TRAb 1.12 U/L (negative < 1.22); TSI antibodies < 0.10 IU/L (0–0.10)] led to methimazole initiation (5 mg daily) thirty‐eight months after amiodarone suspension (Table 1). A subsequent ^99m^Tc‐pertechnetate scintigraphy (forty‐three months after amiodarone suspension) demonstrated a diffuse increased uptake, consistent with type 1 AIT (Figure 4). At the time of the scan, biochemical assessment revealed TSH 0.30 mUI/L and fT4 1.58 ng/dL (Table 1).

## 4 Results

The patient presented a gradual recovery of thyroid function until normalization with methimazole 5 mg daily, which prompted a dose reduction to 2.5 mg daily (Table 1). She remains clinically euthyroid under regular follow‐up in Endocrinology consultations.

## 5 Discussion

This case demonstrates the complete spectrum of amiodarone‐induced thyroid dysfunction (AITD) in a single patient, from severe AIH with myxedema coma to late‐onset type 1 AIT after drug withdrawal.

AIH develops in approximately 5 to 20% of patients receiving amiodarone, occurring more frequently in iodine‐replete areas, and typically occurs within the first six to twelve months of therapy [1, 13]. Female sex, older age, high amiodarone doses (greater than 200 mg/day), cyanotic heart disease, and underlying autoimmune thyroiditis increase susceptibility, some of which were present in our patient [1, 13, 14]. Its pathogenesis is multifactorial and includes iodine‐induced inhibition of thyroid hormone synthesis, reduced peripheral conversion of T4 to T3, and failure to escape the Wolff–Chaikoff effect, which usually resolves within approximately four weeks [13, 14]. AIH may present as either subclinical or clinical, and symptoms are the same as those present in hypothyroidism of other causes, although it may predispose to life‐threatening arrhythmias and acute renal failure [1, 13]. The condition does not usually require amiodarone suspension if the drug is essential for the underlying cardiac disease, as it may be easily managed with levothyroxine supplementation [1, 13]. Myxedema coma in this setting is exceedingly rare, but it has been reported in some studies. A recent systematic review described twelve cases of amiodarone‐induced myxedema coma reported between 2016 and 2022, all over 65 years and mostly females; most patients did not present previous thyroid dysfunction, and most were treated with 200 mg of amiodarone therapy daily [15]. Reported latency between therapy initiation and myxedema coma varied widely, from as early as 12 days to as late as 14 years [15]. Although uncommon, delayed presentation is plausible given amiodarone's prolonged tissue storage. Consistent with this, our patient—without known prior thyroid dysfunction—developed myxedema coma nine years after initiating therapy, similar to a case reported by Raeouf et al. involving a 71‐year‐old patient diagnosed fourteen years after amiodarone initiation [16]. Myxedema coma diagnosis is challenging especially in elderly patients due to non‐specific symptoms, but should be promptly managed with levothyroxine and glucocorticoids (considering adrenal insufficiency possibility) to prevent unfavorable outcomes, as illustrated by our patient's favorable recovery. While amiodarone suspension is not required in AIH, it was possible in this case after proper Cardiology assessment.

AIT develops in around 15% of treated patients and encompasses two major forms. Type 1 AIT is a type of iodine‐induced hyperthyroidism, typically associated with underlying thyroid abnormalities (multinodular goiter or latent Graves' disease); it usually manifests early, with a median onset of around three months after therapy initiation [1]. Type 2 AIT, which is more prevalent, consists of a destructive thyroiditis due to cytotoxic effects of amiodarone that usually develops in an otherwise normal thyroid gland, most common in iodine‐sufficient areas, and which can occur long after amiodarone initiation (median of 30 months) [1, 17]. A third type is also recognized when there is an overlap, and it is difficult to differentiate between the other two types [1]. Clinical AIT presentation is similar to that seen in thyrotoxicosis of other causes, and this condition may exacerbate underlying cardiac conditions, especially in elderly patients [1]. Management differs according to type: type 1 AIT is usually managed with antithyroid drugs and may require definitive treatment in some cases to ultimately solve the thyrotoxicosis, while type 2 AIT is typically self‐limited and manageable with oral glucocorticoids [1]. Contrary to AIH, there is no consensus regarding the decision to either continue or stop amiodarone in AIT; this should be individualized case‐by‐case in a multidisciplinary approach, including Cardiology specialists [1].

In our patient, AIT developed without the classical clinical manifestations of thyrotoxicosis, long after amiodarone suspension. The patient had been treated with levothyroxine supplementation for AIH, with progressively decreasing requirements until therapy was withdrawn; nevertheless, biochemical thyrotoxicosis with negative thyroid autoantibodies persisted even afterwards. Exposure to iodinated supplements/contrasts, corticosteroids, or other thyroid‐interfering medications or supplements was also excluded. Although the delayed onset and absence of underlying thyroid disease would make type 2 AIT more likely, the second ^99m^Tc‐pertechnetate scan showed diffusely increased uptake—suggestive of type 1 AIT—and excluded autonomous nodular activity. The earlier pertechnetate scan likely reflected residual amiodarone effects on iodine handling and uptake pattern, which can persist up to 2 years after withdrawal, as highlighted in the nuclear medicine report. Additionally, despite the fact that urinary iodine concentration was not obtained, it is important to note that Portugal has been identified as a region of moderate iodine deficiency among adults [18]. The massive iodine load from previous long‐term amiodarone therapy—stored in adipose tissue and slowly released—likely acted as a substrate for the Jod‐Basedow effect in a previously iodine‐deficient environment, leading to late‐onset thyrotoxicosis. Moreover, thyroid function's improvement in response to methimazole initiation also favors the diagnosis of type 1 AIT.

Tomisti et al. reported that thyrotoxicosis after amiodarone withdrawal occurred in approximately 5% of patients with type 1 AIT (median onset 6.5 months) and more commonly in those with type 2 AIT (around 20%) [17]. Thyroid volume and type of AIT were confirmed to be the main independent factors influencing the onset time of thyrotoxicosis in this study; the authors also proposed that unusually delayed onset in some type 1 cases may reflect an accompanying destructive process [17]. Our case is noteworthy because it demonstrates a sequential dual‐phase pattern of AITD, which is seldom documented.

Reports of both AIH and AIT occurring sequentially in the same patient are rare and most often reflect a thyroiditis process, evolving from a destructive transient thyrotoxic phase to hypothyroidism as the glandular stores are depleted and the follicles undergo repair [19]. In some instances, this hypothyroidism can become permanent if the underlying damage is extensive [19]. Shazatul Reza et al. described a case of a 53‐year‐old man initially treated for an assumed type 2 AIT and who developed overt hypothyroidism subsequently [10]. McElwaine et al. reported a similar evolution in a 43‐year‐old man treated for type 2 AIT who subsequently required levothyroxine initiation after developing overt hypothyroidism six months later [11]. In both cases, thyroid uptake scans showed absent iodine uptake, and thyroid autoimmunity was negative.

Our case differs in that the patient initially presented with severe AIH culminating in myxedema coma, and only much later developed AIT—an inverse sequence of thyroid dysfunction. This same sequence of events is described by Aleksić et al. in a 66‐year‐old male patient under amiodarone therapy who developed subclinical hypothyroidism after one year of therapy, spontaneously switching to asymptomatic type 2 AIT two years after amiodarone initiation, and in another 46‐year‐old male patient who developed AIH during therapy, followed by symptomatic type 2 AIT eight months after drug withdrawal [20, 21]. Martin‐Du Pan et al. also identified the sequential occurrence of amiodarone‐induced hypothyroidism followed by thyrotoxicosis [22]. Yagishita et al. performed a retrospective analysis of 71 patients who discontinued amiodarone therapy and similarly identified late AIT after drug discontinuation in 7% of patients (median of 11 ± 3 months), all classified as type 2 AIT and all with preceding AIH during therapy, longer treatment duration, and higher cumulative drug exposure—factors proposed to contribute to delayed AIT [9]. Notably, none of these reports describes late‐onset type 1 AIT, as observed in our patient, which does not reflect a cycle of destruction and recovery, but rather a shift in the gland's handling of the iodine load, as the iodine stores seemingly ceased to inhibit the gland and instead began to fuel hormonal synthesis.

## 6 Conclusions

In summary, this case illustrates a rare full‐spectrum presentation of AITD, ranging from myxedema coma to delayed‐onset thyrotoxicosis. Despite absent prior thyroid disease, negative thyroid autoimmunity, and the long interval since amiodarone withdrawal (thirty‐two months), diagnostic imaging and therapeutic response were consistent with type 1 AIT, in contrast with most published cases in which late AIT—even after previous AIH—corresponds to type 2. Long‐term follow‐up will be required to determine whether spontaneous remission or recurrence occurs in our patient. Our report underscores three key points: (1) the prolonged metabolic effects of iodine load and tissue toxicity even after drug cessation, (2) the possibility of reversible AIH preceding subsequent thyrotoxicosis, and (3) the need for long‐term thyroid function monitoring, even after amiodarone withdrawal. Further studies are needed to clarify the mechanisms underlying very late‐onset AIT, particularly type 1, to improve risk stratification and follow‐up strategies.

## Author Contributions

**Daniela M. Soares:** conceptualization, data curation, investigation, writing – original draft. **Lia Ferreira:** conceptualization, supervision, validation, writing – review and editing.

## Funding

The authors have nothing to report.

## Ethics Statement

As a single‐case report with the patient's signed consent, no other ethical review was required.

## Consent

Written informed consent was obtained from the patient for the publication of this case report.

## Conflicts of Interest

The authors declare no conflicts of interest.

## Data Availability

The data used in this article are available upon request from the authors.
